# Plasmablastic Lymphoma presenting as small intestinal polyposis: A case-report

**Published:** 2012-10-30

**Authors:** A Bahari, M Jahantigh, A Mashhadi, Z Bari, AR Bari

**Affiliations:** 1 Department of Gastroenterology, Mashhad University of Medical Sciences, Zahedan, Iran; 2 Department of Pathology, Zahedan University of Medical Sciences, Zahedan, Iran; 3 Department of Hematology and Oncology, Zahedan University of Medical Sciences, Zahedan, Iran; 4 Department of Internal Medicine, Zahedan University of Medical Sciences, Zahedan, Iran; 5 Department of Hematology and Oncology, Shariati Hospital, Tehran University of Medical Sciences, Tehran, Iran

**Keywords:** Plasmablastic lymphoma, Small intestine, Polyposis

## Abstract

**Background:**

Plasmablastic lymphoma (PBL) is a relatively new entity, classified by WHO as a rare variant of diffuse large B cell lymphoma. The present case report introduces a 17 year old girl with chronic diarrhea, abdominal pain, intra-abdominal venous thromboses, ascites, mesenteric lymphadenopathies and small intestinal polyposis, the pathologic and immunohistochemistric examinations of the polypoid lesions were in favor of PBL. Numerous cases of PBL have been reported, but to our knowledge, this is the first report of PBL presenting as small intestinal polyposis.Among lymphomas, only mantle cell lymphoma and follicular cell lymphoma have been previously reported to cause intestinal polyposis. This report introduces Plasmablastic lymphoma, a rare variant of diffuse large B cell lymphoma, as a possible cause of small intestinal polyposis.

## Case Report

A 17 year old girl with a history of 8-year diarrhea presented with abdominal pain and distention. Her bowel movements had been loose and large in volume, occurring 4-5 times a day, containing no blood or mucus and sometimes making her to wake up for defecation at midnight.

Three months before admission in our hospital, but she referred to another hospital due to abdominal pain and distention, but was self-discharged very soon, without reaching to diagnosis. The only documentations which she brought to us, were a report of an abdominopelvic ultrasonography showing normal liver, biliary tract, spleen, kidneys, ovaries and uterus, but accumulation of ascitic fluid and a suspicious intra-abdominal mass, maximally measuring 24mm in diameter and also, a report of a doppler ultrasonography indicating portal vein and superior mesenteric vein thromboses.

When she referred to our hospital, she was still complaining of abdominal pain and diarrhea. She had lost her appetite but mentioned no weight loss due to edema. She sometimes felt low grade fever without chills or night sweat.

She also complained of amenorrhea. Her first menstrual cycle was at the age of 11 years and then she experienced just 2 other menstrual cycles, the last one being 1 year ago.

She was the 5th child in a family of 6 and was uneducated and single. No other member of her family had a history of chronic diarrhea or any significant disease. She mentioned no close contact to a person with tuberculosis. She did not smoke or use alcohol. Her only medication had been warfarin since 2 months before.

On physical examination, her vital signs were stable. She was conscious, but seemed ill. There were multiple freckles on her tanned face skin but not around her lips or on her buccal mucosa. Also, no aphtous lesion was found in her oral cavity.

Her conjunctiva was pale. There was no temporal wasting. She did not have thyromegaly or distended jugular veins. Also no peripheral lymphadenopathy was found. Her breasts were retarded in growth and she had just breast buds. There was no axillary hair. Her heart and lungs were normal on examination. Her abdomen seemed distended. There was no caput medusa or spider angioma. Her bowel sounds were hyperactive.

She had shifting dullness in her abdomen, suggesting the presence of ascitic fluid, but no mass or hepato/splenomegaly was detected. She had no pubic hair. Her extremities were mildly edematous and clubbing was obvious on her fingers. Laboratory data are shown in table-1.

**Table 1:** The patient's laboratory data during admission in our hospital

Test	During admission in our center	Units
White Blood Cell (WBC)	9.6	4-10 (×1000/mL)
Hemoglobin	6.6	12-16 (g/ dL)
Mean Corpuscular Volume	62	80-100 (fL)
Platelet	737	150-450 ( /mL )
Erythrocyte Sedimentation Rate	74	<20 (/ 1st hour)
Blood Urea Nitrogen	0.7	0.6-1.3 (mg/dL)
Potassium	3.1	3.5-5.5 (mg/dL)
Serum Albumin	2.8	3.5-5.3(g/dL)
Prothrombin Time	13.3	11-13 (sec)
Partial Thromboplastin Time	35	30-45 (sec)
Aspartate-amino Transferase	24	0-41 (U/L)
Alanine-amino Transferase	30	0-37 (U/L)
Alkaline Phosphatase	1187	64-306 (U/L)
Lactate Dehydrogenase (LDH)	593	225-500 (U/L)
Iron	18	50-150 μg/dL
TIBC	210	300-360 μg/dL
Ferritin	11	50-200 μg/L
Stool examination	normal	
Serum-ascitic albumin gradient	2.5	(g/dL)

She went under upper endoscopy and biopsy from the 2nd part of the duodenum. It grossly revealed hypertensive gastropathy without varix formation, but pathologic examination of the biopsy specimen showed villous atrophy with crypt hyperplasia and severe lymphocytic infiltration. Tests for anti-endomysial IgA, anti-gliadin IgG and anti tissue transglutaminase (anti tTG) IgA and IgG had negative results.

**Fig (1AB):**
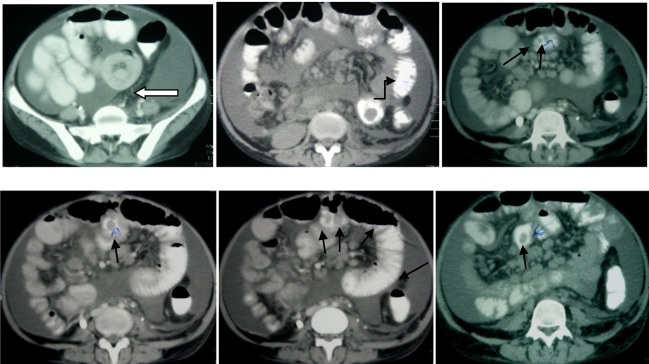
The patient's abdominopelvic CT scan showing the mass causing a non-obstructing intussusceptions (hollow arrow) and pedunculated polypoid lesions of the small intestine (black arrows).

Colonoscopy was also performed, revealing normal macroscopic appearance of mucosa, but no biopsy was taken. An abdominopelvic spiral CT-scan was also performed, showing multiple mesenteric lymphadenopathies and multiple pedunculated polypoid lesions in her small intestine. There was also an oval shaped mass in ileo-jejunal area measuring 60x40mm, causing a non-obstructing intussusception (Figure.1).

**Fig. 2:**
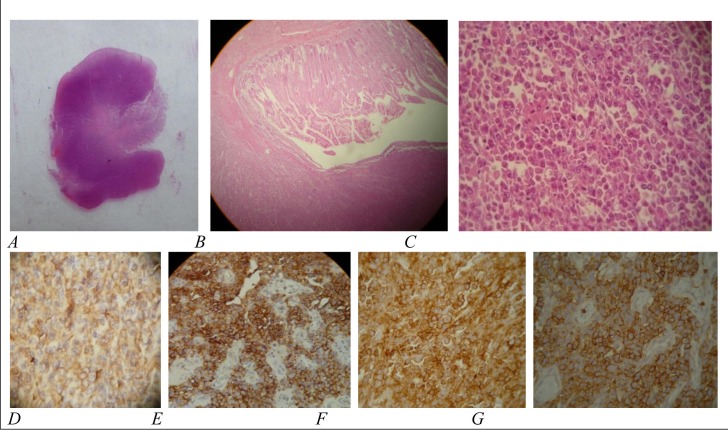
Pathologic and IHC examination of a small intestinal polyp: A) gross view of a prepared section of a pedunculated polyp, B) infiltration of lympho-plasmacytoid tumoral cells (original magnification ×10), C) discohesive medium to large sized lympho-plasmacytoid neoplastic cells with large vesicular nuclei, prominent nucleoli, scant to moderate eosinophilic cytoplasm and numerous mitotic figures with atypical forms (hematoxylin and eosin, original magnification ×40), D-G) immunoreactivity of tumoral cells for CD79 (figure D), CD138 (figure E), epidermal membrane antigen (figure F) and leukocyte common antigen (figure G) (original magnification ×40).

At this time, she underwent laparatomy for excisional biopsy of the lymph nodes and resection of the mentioned masses. During the operation, multiple polypoid lesions were found in the small intestinal lumen by touching. One of these polypoid lesions had caused a non-obstructing intussusception at about 100 cm distance from the beginning of the jejunum. Multiple mesenteric lymphadenopathies were also found, but there was no retroperitoneal mass. The intussuscepting mass with 2 adjacent polyps and some lymph nodes were excised for evaluation. Histopathologic examination showed polypoid lesions with ulcerated surface epithelium and destroyed glandular architecture due to diffuse infiltration of discohesive medium to large sized lympho-plasmacytoid neoplastic cells. The tumoral cells had large vesicular nuclei, prominent nucleoli and scant to moderate eosinophilic cytoplasm. Mitotic figures with atypical forms were numerous (Figure-2). All of the excised lymph nodes were also involved by neoplastic cells. The histopathologic fnidings were consistent with lymphoma. The specimens were also evaluated using Immuno-histo-chemical staining. The panel of IHC included leukocyte common antigen (LCA), epidermal membrane antigen (EMA), CD3, CD20, CD79, CD30, CD138, anaplastic large cell lymphoma kinase 1 (ALK 1) and melan A (all from Novocastra). The tumor cells expressed LCA and were positive for EMA, CD138 and CD79, but were negative for CD3, CD20,CD30, ALK 1 and melan A (Figure-2).

All together, the histologic and IHC results were in favor of plasmablastic lymphoma (PBL), but before starting chemotherapy, unfortunately the patient died.

## Discussion

In our patient, the presence of chronic diarrhea accompanied by anemia, hypoalbuminemia, delayed puberty and amenorrhea were suggestive of malabsorption.

Iron deficiency anemia in the absence of menstrual cycles necessitated the evaluation of GI tract. Although duodenal biopsy showed lymphocytic infiltration with villous atrophy, these findings were not specific and could be seen in celiac sprue, bacterial overgrowth, crohn s disease, cow s milk protein intolerance (in children), eosinophilic gastroenteritis, giardiasis, lymphoma, peptic duodenitis, tropical sprue, common variable immunodeficiency, autoimmune enteropathy and other immunodeficiency states.(1)

Negative antiendomysial and antigliadin and anti-tTG tests ruled out celiac sprue. In order to search for the other mentioned causes, colonoscopy and evaluation of small intestine were needed.

On the other hand, the presence of high gradient ascites in the absence of heart failure, nephrose or thyroid disease led to ultrasonographic evaluation of the liver and spleen, which subsequently showed intra-abdominal venous thromboses. So, the high gradient ascites seemed to be due to portal hypertension caused by the thromboses. Here, the explanation for the companion of malabsorption and thrombosis could be hyperhomocysteinemia due to folic acid deficiency, (2) extraintestinal complication of inflammatory bowel disease, (3) secondary to the presence of an obscure GI malignancy(4) or even thrombosis due to an underlying mass effect.

All together, the etiology of malabsorption had been still undiagnosed until the observation of intraabdominal lymphadenopathies and small intestinal polypoid lesions led to laparatomy and final diagnosis of lymphoma.

The unique feature of our case was presentation of PBL as small intestinal polyposis. The common small intestinal polyposis syndromes include juvenile polyposis,(5) Peutz-Jeghers syndrome,(5) neurofibromatosis(5) and the less common ones include Cronkhite-Canada syndrome(6) and Cowden syndrome (7) and among non-hudgkin lymphomas, only mantle cell lymphoma (lymphomatous pilyposis) and some cases of follicular lymphoma of the GI tract have been reported with multiple polypoid appearance.(8-10)

PBL is a relatively new entity, classified by WHO as a rare variant of diffuse large B cell lymphoma,(11-12) initially described by Delecluse et al in 1997.(13) It is commonly associated with acquired immunodeficiency syndrome (AIDS), with predilection for the mucosa of oral cavity.(14)

Numerous cases of PBL presenting as buccal mass, cervical lymph node, lung tumor, isolated cutaneous mass, nasopharyngeal lesion, gastric tumor or even after therapy for inflammatory bowel disease, post solid organ transplantation or as a variant of Richter's syndrome have been reported,(12-25) but to our knowledge, this is the first report of PBL presenting as small intestinal polyposis.

According to the pathologic and IHC evaluation, the most important differential diagnoses to be ruled out were anaplastic plasmacytoma/multiple myeloma and anaplastic large cell lymphoma. The immunophenotype of anaplastic plasmacytoma/ multiple myeloma may be similar to that of PBL, but bone marrow involvement is a common finding. Furthermore, the neoplastic cells tend to have a larger cytoplasm and eccentrically located nuclei(18) Negative ALK-1, CD30 and positive CD138 were inconsistent with the diagnosis of anaplastic large cell lymphoma. Besides, tumor cells in anaplastic large cell lymphoma tend to be pleomorphic and bizarre and also plasmacytoid differentiation is not common.(18) Therefore, according to plasmacytoid differentiation, negative CD20 as a conventional B cell marker and strong immunoreactivity for CD138 as a marker of plasmacell differentiation, the diagnosis of PBL was established.

However, due to the aggressive behavior of PBL, the patient's chronic diarrhea could not be explained. Since PBL commonly occurs in immunodeficient patients, especially in association with HIV infection, immunodeficiency could have been the underlying reason for her long lasting diarrhea, but the patient died before the diagnosis of PBL was made. There fore, no test for HIV infection could be performed. It is also possible that another underlying cause, which we could not find, had been responsible for her long lasting diarrhea.

In conclusion, lymphoma should be kept in mind as a cause of small intestinal polyposis. Also, beside mantle cell and follicular lymphomas, this report introduces PBL as a rare but possible cause of small intestinal polyposis.
